# Enhancing the Fruit Yield and Quality in Pomegranate: Insights into Drip Irrigation and Mulching Strategies

**DOI:** 10.3390/plants12183241

**Published:** 2023-09-12

**Authors:** Ranjitha Beelagi, Vijay P. Singh, Rajkumar Jat, Pramod Kumar Singh, Ratna Rai, Akath Singh, Boris Basile, Alessandro Mataffo, Giandomenico Corrado, Pradeep Kumar

**Affiliations:** 1 Department of Horticulture, College of Agriculture, G. B. Pant University of Agriculture and Technology, Pantnagar 263145, India; 2021ranjitagombe@gmail.com (R.B.); rajrulez95@gmail.com (R.J.); ratnarai1975@gmail.com (R.R.); 2 Department of Irrigation and Drainage Engineering, College of Technology, G. B. Pant University of Agriculture and Technology, Pantnagar 263145, India; singhp67@gmail.com; 3 Division of Integrated Farming System, ICAR—Central Arid Zone Research Institute, Jodhpur 342003, India; akath.singh@icar.gov.in (A.S.); pradeep.umar4@icar.gov.in (P.K.); 4 Department of Agricultural Sciences, University of Naples Federico II, 80055 Portici, Italy; boris.basile@unina.it (B.B.); alessandro.mataffo@unina.it (A.M.)

**Keywords:** fruit quality, drought, yield, water-saving technologies, tree, *Punica granatum*, sustainable agriculture

## Abstract

Pomegranate (*Punica granatum* L.) is a fruit tree that is globally distributed, especially in warm areas with low annual rainfall and limited water availability. This species exemplifies the critical role of water in agriculture and the need for efficient irrigation practices due to its characteristics, cultivation requirements, and geographic diffusion. In this study, we investigated the effects of drip irrigation and mulching on the vegetative growth, yield, and fruit quality attributes of pomegranate. The experiment involved three irrigation regimes (100% of evapotranspiration, 80%, and 60%) and three mulching treatments (no mulch, plastic mulch, and organic mulch) in a factorial combination. Both irrigation and mulching had significant positive influences on the yield and fruit quality attributes. Specifically, deficit irrigation strategies showed a negative impact on the fruit yield per tree, with a greater effect observed as the severity of the irrigation deficit increased. Mulching, on the other hand, led to a significant increase in the fruit yield, primarily attributed to an increase in fruit size. Furthermore, the analysis indicated that irrigation and mulching treatments had distinct effects on fruit traits such as the fruit length, width, volume, and rind thickness. Interestingly, the study highlighted that the effects of irrigation and mulching on fruit quality attributes were mostly independent of each other, suggesting an additive influence rather than an interaction between the two factors. These findings underscore the importance of considering irrigation and mulching practices for optimizing fruit quality in pomegranate cultivation, particularly in semi-arid regions. The results contribute valuable insights for farmers and researchers seeking to enhance fruit production and quality.

## 1. Introduction

Pomegranate (*Punica granatum* L.) is a tropical and subtropical fruit gaining popularity due to its potential health benefits [1,2]. This tree has the potential to significantly enhance the quality of the dietary basket in countries rich in diverse indigenous flora, contributing to sustainable food practices [3]. Pomegranate is cultivated in various regions, including Asia, the Mediterranean basin, and the United States [4], with India leading in pomegranate production, contributing to nearly half of the global output [5,6]. Pomegranate requires regular irrigation due to their shallow root system, primarily confined to a depth of 45–60 cm in light soils [7]. In India, conventional surface irrigation methods are commonly employed for field crops, resulting in significant water loss due to evaporation, runoff, seepage, and percolation [8]. Therefore, there is a need to expand our scientific understanding of the effects of various agronomic tools and their possible synergistic action that can increase the efficacy of water supply [9,10]. The combination of climate change, water restrictions during droughts, and the expansion of pomegranate cultivation in semi-arid regions forces farmers to effectively manage increasingly limited and lower-quality water resources [11,12,13].

Among conventional irrigation approaches, drip irrigation (DI) is widely considered one of the most effective methods for achieving efficient water use in large-scale horticulture [14,15]. This method provides the frequent, slow, and precise application of water directly to the plant root zone (subsurface DI) or the soil surface around the plant (surface DI). Leaving aside economic considerations, DI, when combined with mulching, forms a powerful water-saving technology in agriculture. Mulching refers to the practice of covering the soil surface around plants with a protective layer of organic or inorganic materials [16,17], such as straw, wood chips, bark, plastic films, and compost [18]. Mulch helps to conserve soil moisture by reducing evaporation, prevent weed growth by blocking sunlight, regulate soil temperature, and protect the root system from extreme temperatures [16,17,19].

DI under mulch has been successfully implemented across various crops in semi-arid and arid regions. The evaluation of two mulching systems (transparent and non-transparent), along with the integration with DI, was conducted on ten important herbaceous annual crops in Lebanon [20]. Mulching significantly reduced the water footprint of all crops and, when combined with the substitution of conventional irrigation methods (such as surface or sprinkler irrigation) with DI, an additional reduction in water footprint was observed for most crops [20]. The study also indicated that the effect on crop production was more influenced by climate conditions rather than the soil type [20]. In Syria, transparent mulch in combination with DI resulted in the highest cucumber yield and water use efficiency. This condition outperformed other treatments, including black mulch with DI, DI without mulching, and surface furrow irrigation [21]. Moreover, the transparent mulch resulted in higher soil temperatures and moisture levels throughout the growing season (compared to the black mulch treatment), which may have promoted the emergence and vegetative growth of cucumber [21]. Film mulching can also help to increase soil temperature, and for instance, ensured a high emergence ratio in maiz [22]. Moreover, DI offered significant advantages by increasing the water and fertilizer efficiency. Specifically, among the treatments, the best results in terms of the yield, nitrogen efficiency, and overall economic benefits were obtained with the combination of mulching and DI [22]. In subtropical conditions, fully irrigated mulched guava (*Psidium guajava* L.) showed the highest vegetative growth and leaf nutrient contents. However, using deficit irrigation at 75% of the evapotranspiration rate with plastic mulch during the fruit growth period increased the fruit yield [23].

Previous studies have also shown the positive effects of combining DI with mulching on the growth, yield, and water use efficiency on fruit trees typically found in regions with warm to hot climates. Evidence for the superior performance of DI combined with mulching was, for example, found in the semi-arid Terai region of India for high-density young plantations of the subtropical tree litchi (*Litchi chinensis* Sonn., Sapindaceae). Among various treatment combinations involving different water restitutions and the presence or absence of mulching, the approach of applying DI at 100% of the water requirement alongside mulching emerged as the most effective [24]. Conversely, DI at 50% without mulch application was considered the least efficient [24]. In mango (*Mangifera indica* L.) cultivated in the Teragana region of southern India, applying 100% of the crop’s evapotranspiration (ET_c_) along with mulching resulted in the highest fruit yield [25]. However, the highest water use efficiency was observed with 75% ET_c_ with mulching, without a negative effect on fruit quality [25]. In arid conditions, the indirect subsurface DI increased the surface wetted area and soil water storage and decreased evaporation in a jujube (*Ziziphus jujuba* Mill.) plantation with plastic mulch [26].

Given the economic importance of pomegranate and the reliance on water management, the aims of this study were to investigate the effects of different mulching types and water regimes using DI. Specifically, the study aimed to assess the impact of the absence or presence of either organic or plastic mulch and three DI regimes on the plant growth, yield, and fruit quality parameters. The goal was to provide valuable insights into the potential benefits of mulching and water management strategies for ensuring pomegranate crop performance and enhancing sustainability.

## 2. Results

### 2.1. Vegetative Growth

The experimental work encompassed a factorial combination of three distinct irrigation treatments and three mulching treatments. The irrigation treatments comprised two deficit irrigation doses, 80% (I80) and 60% (I60) of the calculated water requirement (WR), in addition to a fully irrigated control (I100), receiving 100% of the WR. The three mulching treatments included plastic mulch (PM), organic mulch (OM), and an un-mulched control (NoM). Prior to the application of the experimental treatments (time 0, t_0_), there were no discernible differences observed in any of the measured plant vegetative traits, namely, the plant height, canopy circumference, and tree volume (Table 1). This finding confirmed the initial homogeneity of the selected plant material for the trial. However, at the conclusion of the experiment (t_end_), both the irrigation and mulching treatments exhibited significant effects on these vegetative parameters, while the interaction between irrigation and mulching did not have a notable impact on these traits (Table 1). Specifically, the I60 treatment resulted in a 6% decrease in the plant height and canopy circumference, along with a 17% reduction in tree volume compared to the other two irrigation treatments (I100 and I80). On the other hand, the implementation of plastic mulch significantly increased the plant height, canopy circumference, and tree volume by approximately 4%, 6%, and 16%, respectively, compared to the NoM and OM treatments, although there was no significant difference in plant height between NoM and OM trees (Table 1).

### 2.2. Yield Components and Fruit Quality at Harvest

Both deficit irrigation conditions (I80 and I60) exhibited a negative impact on the fruit yield per tree compared to fully irrigated plants (I100) (Table 2). The adverse effect on the fruit yield intensified when transitioning from I80 to I60, resulting in a decrease of 12% and 24%, respectively. The data indicated that this decline in the fruit yield under deficit irrigation can be attributed to a reduction in both yield components measured during the study (Table 2). Furthermore, regardless of the irrigation treatment, the application of mulching demonstrated a significant increase in the fruit yield, averaging around 30% compared to non-mulched (NoM) plants. This increase in the fruit yield can be mainly attributed to a significant enhancement in the fruit size (averaging 22%), while there was a slight increase in the number of fruits per tree (averaging 6%).

**Figure 1:**
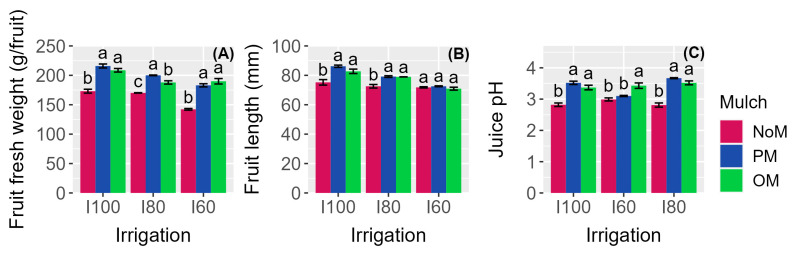
Effect of the irrigation and mulching treatments on the (**A**) fruit fresh weight, (**B**) fruit length, and (**C**) juice pH at harvest. Within each panel and separately for each irrigation treatment, means followed by a common letter are not significantly different according to the Tukey test (*p* ≤ 0.05).

Among the yield parameters examined, it was found that the interaction between the irrigation and mulching treatments had a significant effect on the fruit fresh weight (Table 2, Figure 1A). Specifically, mulching increased the fruit fresh weight compared to the NoM treatment, but the intensity of this effect depended on the irrigation treatments, resulting in it being stronger in the most severe deficit irrigation treatment (+31% in I60 trees) compared to I80 plants (+14%) and fully irrigated trees (+22%) (Figure 1A).

The fruit length was found to be influenced by the irrigation regime, the mulching, and their interaction. The results presented in Table 3 and Figure 1B indicate that the fruit length progressively decreased as the severity of the deficit irrigation increased from I100 to I60. Mulching had a positive effect on the fruit length, but this effect was only significant in I100 and I80 trees. In I60 plants, the effect of mulching on the fruit length was not significant. On the other hand, the fruit width was affected solely by the irrigation treatments. Specifically, the fruit width was 8% smaller in I60 trees compared to in I100 trees (Table 3). The mulch treatments did not have a significant effect on the fruit width. Regarding the fruit volume, a clear trend was observed, where it decreased with the increasing severity of deficit irrigation. Compared to I100 trees, the fruit volume decreased by 13% in I80 trees and by 26% in I60 trees. Mulching, however, had a positive impact on the fruit volume, as it increased compared to NoM plants, with an average increase of 18%. The fruit shape index, which provides insights into the elongation/roundness of the fruit and its marketability, was not significantly influenced by either the irrigation or mulch treatments, nor their interaction (Table 3). The rind thickness, another important commercial parameter, was slightly smaller in I60 trees compared to that in the other irrigation treatments. However, the application of mulch increased the rind thickness compared to NoM trees, with an increase of 24% in OM-treated trees and of 36% in PM-treated trees (Table 3).

The total soluble solids, total sugars, and reducing sugars in the fruit juice at harvest were significantly lower in I60 trees compared to those in I80 and I100 trees, showing reductions of approximately 4%, 4%, and 5%, respectively (Table 4). Conversely, both mulching treatments led to an increase in these parameters compared to NoM trees (Table 4). No significant effect of the I × M interaction was observed on these fruit qualitative traits (Table 4). The juice pH was significantly influenced by both the irrigation and mulching treatments, as well as by the I × M interaction (Table 4; Figure 1C). When comparing I100 and I80 trees, the juice pH was significantly higher when mulching was applied compared to NoM trees. In the case of I60 trees, the juice pH was higher in OM-treated trees compared to that in PM and NoM trees (Figure 1C). The TSS/TA ratio of the fruit juice at harvest was significantly higher in I80 trees compared to that in I60 and I100 trees. Furthermore, the application of mulching treatments induced a substantial 36% increase in this parameter compared to that in NoM trees (Table 4).

### 2.3. Multivariate Analysis

The principal component analysis extracted eight principal components (Dim.; Supplementary Table S1), out of which the first three exhibited eigenvalues exceeding one. These three components accounted for a cumulative variance of 93% of the total. Dim. 1 showed a positive correlation with several variables including the plant height at t_end_, canopy circumference at t_end_, tree volume at t_end_, number of fruit per tree, fruit yield per tree, fruit fresh weight, fruit length, fruit width, fruit volume, rind thickness, fruit shape index, juice pH, juice total soluble solids (TSS), juice TSS/TA ratio, juice total sugar, and juice reducing sugars. Conversely, Dim. 1 displayed a negative correlation with the juice titratable acidity (TA). Dim. 2 demonstrated a positive correlation with the canopy circumference at t_0_ and tree volume at t_0_, while Dim. 3 displayed a positive correlation with the plant height at t_0_.

**Figure 2:**
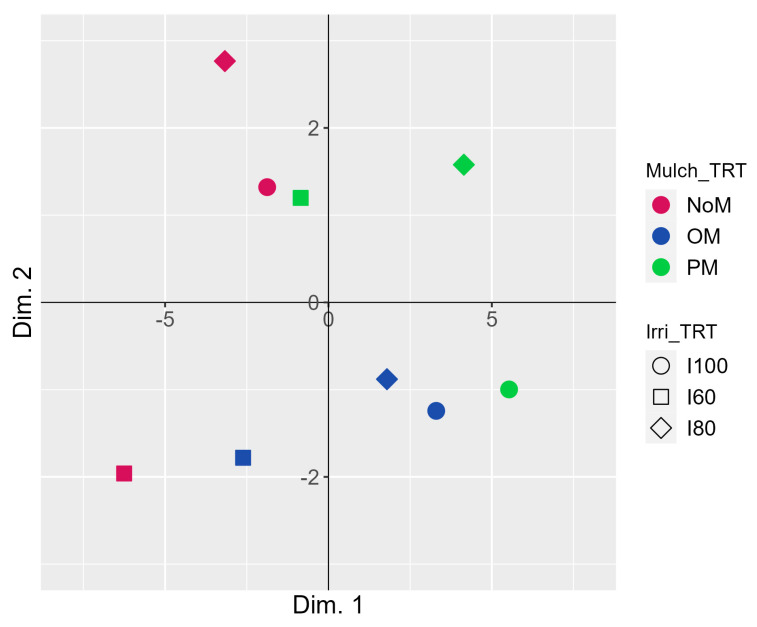
PCA plot of the experimental conditions under investigation according to the type of mulching (Mulc_TRT) and level of irrigation (Irri_TRT). The PCA plot illustrates the distribution and relationships of samples based on the measured variables. Shapes represent different irrigation treatments, while colors represent different mulching treatments, as indicated on the right-hand side of the plot. Dim. 1 and 2 accounted for 67.80% and 13.15% of the total variance, respectively.

The bidimensional PCA plot provides a visual representation of the similarities and differences among treatments and the overall relationships between irrigation, mulching, and their effect on the measured variables (Figure 2). 

Experimental treatments are rather uniformly distributed in all quadrants, suggesting a diverse response across the conditions under investigation. This implies that the variation observed in the data is relatively homogeneous and not strongly influenced by a single treatment (irrigation and mulching). Moreover, the absence of clear clustering (e.g., according to each factor and their levels) indicates that both irrigation and mulching treatments are significant in contributing to the observed variations. The distribution patterns on the PCA plot also provide interesting information. The NoM treatment appears to be primarily separated along Dimension 2, while the OM treatment shows separation along Dimension 1. The dominant separation along either axis suggests that different associated variables (Table S1) have a stronger influence on the observed variations for mulching. In the case of the third mulching type (PM), separation is observed along both axes, indicating that with plastic mulch, the variability in the dataset is not primarily driven by one specific variable or factor. Finally, the observation that I100, the fully irrigated treatment, does not exhibit a large influence on the two mulching types aligns with the notion that the impact of the kind of mulching is not as pronounced in the full irrigation regime, and strong differences are relative to the absence of mulching.

## 3. Discussion

In commercial cultivation, pomegranate trees are typically accustomed to receiving regular water supply [4]. Therefore, in this study, we prioritized irrigation treatments that do not induce severe water stress and allow for economic profitability. The irrigation treatment I60 led to a significant inhibition of the plant height and canopy volume compared to the other two irrigation treatments. In pomegranate, a DI with 60% of the evapotranspiration cannot be considered a severe stress, especially if present from the beginning of the season to the end of the first half of the linear fruit growth phase [27]. While there is a large body of literature supporting the notion that irrigation plays a crucial role in the crop vegetative growth [28], several reports on pomegranate focus primarly on fruit yield and quality, probably because these are crucial factors for decision making on irrigation [29]. In pomegranate, a decrease from 15 m^3^ (the highest dose under investigation, compared to 11 m^3^ of the control condition) to 7 m^3^ per tree resulted in a diminished vegetative growth, assessed measuring the shoot length, number of leaves per shoot, and leaf area [30]. Among DI treatments, only the constantly applied 50% of the control throughout the whole growing season treatment showed a substantial decrease in the trunk growth and canopy size [31]. Moreover, negative effects on the tree canopy size were observed when applying irrigation deficits at 35% and 50% of the crop water use [32]. All these findings confirm the importance of providing adequate irrigation to support optimal vegetative growth and canopy development in pomegranate and that water deficits that roughly approximate half of the control condition hinder the overall size and vigor of the trees. On the other hand, the application of plastic mulch increased the plant height, canopy circumference, and tree volume compared to the control treatments, although there was no significant difference in plant height between the trees with plastic or organic mulch. The effect of OM on plant growth was modest and non-significant. Mulching has been widely recognized as an effective agronomic practice for conserving soil moisture, controlling weed growth, moderating soil temperature, and improving overall plant performance [16,33]. Moreover, mulch can also modify soil conditions, promoting nutrient availability, root development, and microbial activity, which ultimately contribute to improved plant growth in sub-optimal conditions [16,33]. Our results imply that, considering vegetative parameters, the choice of the mulching material has significant implications in pomegranate. There are various differences between organic and plastic mulch, with the latter being more efficient in avoiding water evaporation, controlling weeds, and limiting extreme soil temperature, and the former being more sustainable and usually affordable and, in the long term, also able to improve soil fertility and microbial activity [16,33,34]. The lack of interaction of the mulch type with irrigation may suggest that the higher effect on soil temperature and, more generally, the higher insulating properties of plastic mulch could be beneficial for promoting plant vegetative growth—for example, by enhancing the root activity and nutrient uptake. In pomegranate, among black polythene, banana trash, and sawdust, the former was more effective in avoiding extreme soil temperatures and retaining soil moisture [35].

Moving on to fruit yield components, the deficit irrigation strategies (I80 and I60) had a negative impact on the fruit yield per tree compared to fully irrigated plants. The severity of the deficit irrigation treatment was directly related to the decrease in the fruit yield, with I60 showing the most significant reduction. This reduction in the yield was associated with a decrease in all the yield components measured in the study. Interestingly, regardless of the irrigation treatment, the application of mulch significantly increased the fruit yield, mainly due to an increase in the fruit size and, to a lesser extent, the number of fruits per tree. These findings confirmed that deficit irrigation can have adverse effects on the pomegranate yield [27,31], but this negative impact can be mitigated using mulching [36,37]. A significant factor interaction was noted only for fruit length, although this variation did not affect the fruit shape index. Specifically, the fruit length decreased with the increasing severity of the deficit irrigation, but mulching induced a compensatory increase in the fruit length, particularly in fully irrigated and moderately deficit irrigated trees. The fruit width was only affected by the irrigation treatments, with a smaller fruit width observed in the most severe deficit irrigation treatment (I60). The effects of irrigation and mulching on pomegranate production are likely to be cumulative and additive, meaning that they can independently contribute to changes in the overall outcome and complement each other at the fruit morphological level. Irrigation directly affects the water status of the plants, which is essential for productivity, while mulching primarily impacts fruit parameters indirectly, as it acts by altering soil and root zone conditions.

The irrigation treatments significantly influenced all the fruit quality parameters. The TSS, total sugars, and reducing sugars in fruit juice were lower in the most severe deficit irrigation treatment compared to I80 and I100, and coherently, higher acidity and pH values were observed in the I60. Conversely, the application of mulch had the opposite effect regarding water stress compared to non-mulched plants. A difference between the mulching type was observed only for the sugars. These findings emphasize the significance of irrigation and mulching in shaping fruit quality attributes in pomegranate, including sweetness and acidity, two important commercial features. The accumulation of sugars in fruits is influenced by several factors, including genetic factors, environmental conditions, and cultural practices [38,39]. The higher content of reducing sugars suggests an increased fruit maturity, which should act alongside an improved photosynthesis and carbohydrate accumulation, as indicated by the concurrent increased total sugar content [40,41]. As mulching primarily acts on the root system, the source–sink relationship (including hormonal factors), plant nutritional status, and water availability may be the primary causes of these effects [38,42].

The multivariate analysis through dimensional reduction indicated that neither the irrigation nor the mulching treatment appeared to have a dominant or overriding influence on the variance of the measured parameters. The effects of irrigation and mulching on pomegranate growth and development are more likely to be additive, meaning that they independently contributed to the overall plant response. The ANOVA results likely supported this observation by showing that the main effects of irrigation and mulching were statistically significant, while factor interaction was not significant for almost all the parameters. It is possible to hypothesize that, at the physiological level, each factor likely affected the plants in a similar way, and when combined, they contributed to balancing the plant response. These physiological mechanisms could include those related to water availability and stress responses for irrigation [43,44,45,46,47] and changes in soil moisture, temperature, and nutrient availability for mulching [18,48,49,50].

The use of plastic mulch proved to be particularly effective in enhancing the plant height, canopy circumference, tree volume, fruit yield, and fruit quality compared to non-mulched plants. Interestingly, the I80 treatment demonstrates several positive features that make it comparable to the I100 treatment. Especially in a semi-arid zone, a 20% reduction in water use can play a crucial role in sustainable agriculture by preserving the water source and optimizing freshwater management practices while ensuring satisfactory agricultural productivity, besides providing economic benefits. Both organic and plastic mulch can contribute to improved plant growth, an increased fruit yield, and an enhanced fruit quality. The choice between the two types of mulch may depend on various factors that include the availability, cost, and also other effects on agronomic practices that were not analyzed in this study [34]. 

## 4. Materials and Methods

### 4.1. Experimental Site and Plant Material

The experiment was conducted during the 2018–19 growing season at the Horticulture Research Center of the G.B. Pant University of Agriculture and Technology (GBPUAT), Pantnagar, Uttarakhand, India. The pomegranate (*Punica granatum* L.) trees used in the study were 4 years old and of the ‘Bhagwa’ variety. They were spaced at 5.0 m × 3.0 m, resulting in a planting density of 666.7 trees per hectare. Fifty-four healthy, uniform, and vigorously growing plants were selected for the study. At the experimental site, the soil was alluvial with a sandy-clay texture (USDA soil textural triangle; https://www.nrcs.usda.gov/sites/default/files/2022-09/The-Soil-Survey-Manual.pdf; last accessed: 1 July 2023).

The climate at the experimental site is humid subtropical, characterized by dry and hot summers (with air temperatures ranging between 39 and 45 °C) and cool winters (with air temperatures ranging between 0 and 9 °C). The average yearly rainfall at the site is 1200 mm. Throughout the experiment, various microclimatic parameters such as the air temperature, air relative humidity, solar radiation, wind speed, reference evapotranspiration, and rainfall were measured using an automatic weather station located 5 km away from the experimental site (Figure S1). The experimental orchard was managed following standard practices for commercial fruit production, which included pest and disease management. Pruning was conducted on 20 March 2019, and fertilizers were applied at a rate of 500 g of nitrogen (N), 125 g of phosphorus pentoxide (P_2_O_5_), and 250 g of potassium oxide (K_2_O) per tree per year.

### 4.2. Experimental Design

The experimental design employed was a randomized complete-block design with nine treatments and three replications. The nine treatments were created by combining three irrigation and three mulching treatments in a factorial arrangement. The irrigation treatments consisted of two deficit irrigation doses, where 80% and 60% of the calculated water requirement (WR) were supplied through irrigation (referred to as I80 and I60, respectively), and a fully irrigated control that received 100% of the WR (referred to as I100). Trees were irrigated every other day using a drip irrigation system equipped with four compensated emitters (8 L h^−1^) per plant. The WR (in liters per tree) was calculated by applying the following formula cumulatively over two consecutive days.

(1) $$WR={\pi \left(\frac{D}{2}\right)}^{2}\times \frac{{ET}_{c}-E_{r}}{E_{i}}$$

where D is the mean tree canopy diameter (m), ET_c_ (mm) is the calculated crop evapotranspiration cumulated over two consecutive days, E_r_ (mm) is the effective rainfall cumulated over two consecutive days, and E_i_ is the irrigation efficiency of the drip system (0.90). ET_c_ was calculated as follows:

(2) $${ET}_{c}={ET}_{o}\times K_{c}$$

where ET_o_ is the reference evapotranspiration and K_c_ is the crop coefficient (0.7 for no mulching, 0.65 for the organic mulching, and 0.6 for the plastic mulching). ET_o_ was calculated using the following equation:

(3) $${ET}_{o}=E_{p}\times K_{p}$$

where E_p_ is the pan evaporation cumulated over two consecutive days (mm) and K_p_ is the pan coefficient (0.7). All the previous calculations followed the FAO guidelines [51]. Water used for irrigation had an electrical conductivity of 0.4 dS m^−1^.

The three mulching treatments were as follows: plastic mulch (PM), organic mulch (OM), and an un-mulched control (NoM). For the PM treatment, 100-micron sheets of bicolored polyethylene measuring 1.2 m in width (with black on the lower side and a silver color on the upper side) were applied at the base of the trees, and their edges were buried in the soil (requiring 200 g of polyethylene per plant). For the OM treatment, locally collected dried Kans grass (*Saccharum spontaneum* L.) was uniformly applied at the base of the trees to form a 15 cm thick layer (utilizing 17 kg of dry Kans grass per m^2^ of soil surface). Prior to the mulching application, weeds were meticulously removed in all treatments. Mulches were applied after pruning. The nine treatments are therefore denoted as follows: I100-NoM (fully irrigated and un-mulched control), I100-PM (100% WR and plastic mulch), I100-OM (100% WR and organic mulch), I80-NoM (80% WR and no mulch), I80-PM (80% WR and plastic mulch), I80-OM (80% WR and organic mulch), I60-NoM (60% WR and no mulch), I60-PM (60% WR and plastic mulch), and I60-OM (60% WR and organic mulch). During the whole experiment, fully irrigated trees exposed to the different mulching treatments, namely, I100-NoM, I100-OM, and I100-PM, received, with irrigation, 100% of the WR, corresponding to a total amount of water of around 686, 651, and 573 mm, respectively.

### 4.3. Measurement of Vegetative Growth

For each tree included in the experiment, the plant height and two orthogonal canopy diameters (measured in the east–west and north–south directions) were determined using a measuring tape at the beginning of the experiment (t_0_, 15 January 2019) and at the end of the experiment (t_end_, 6 December 2019). The canopy circumference at t_0_ and t_end_ was calculated by assuming its shape as an ellipse, with the measured diameters as its axes. Similarly, the tree volume at t_0_ and t_end_ was calculated by assuming its shape as an ellipsoid, with the canopy diameters and tree height as its axes.

### 4.4. Fruit Harvest, Yield Components, and Fruit Composition

Fruit harvesting was conducted in September 2019. For each tree included in the experiment, the number of fruits and the fruit yield per tree were determined by counting and weighing the fruits individually. A sample of ten fruits per tree was collected and immediately transported to the laboratory for the assessment of fruit quality traits. The length and width of each fruit were measured using a digital caliper (Mitutoyo Digital Vernier Caliper; Mumbai, Maharashtra, India), and the fresh weight was measured using a digital scale (RPM 100, RPM Corporation; Thane, Maharashtra, India). The fruit volume was calculated assuming the shape as an ellipse, with the fruit length and diameter as the axes (considering two axes equal to the fruit diameter). Before juice extraction, each fruit was opened, and the rind thickness was measured with a digital caliper. Fruit juice was used to measure the total soluble solids (TSS), pH, titratable acidity (TA), and total and reducing sugar content by taking five fruits per plant. The total soluble solids were measured using a refractometer (ERMA hand refractometer, Ases Chemical Works, Jodhpur, Rajasthan, India). The titratable acidity (TA) of the fruit juice was determined by adding 0.1N NaOH until reaching a pH endpoint of 8.3. The pH of the juice was measured using a pH meter (LT-10; Labtronics; Panchkula, Haryana, India). These data were used to calculate the TSS/TA ratio. The total and reducing sugars in the fruit juice were measured using the Lane and Eynon method, as previously described [52].

### 4.5. Statistical Analysis

The effects of irrigation (I), mulching (M), and their interactions (I × M) on all the measured parameters were assessed using a two-way ANOVA. The Tukey’s honestly significant difference (HSD) test was used for mean separation (*p* ≤ 0.05). Principal Component Analysis (PCA) utilized 20 original variables as the input, including the plant height at t_0_, plant height at t_end_, canopy circumference at t_0_, canopy circumference at t_end_, tree volume at t_0_, tree volume at t_end_, number of fruits per tree, fruit yield per tree, fruit fresh weight, fruit length, fruit width, fruit volume, rind thickness, fruit shape index, juice pH, juice total soluble solids (TSS), juice titratable acidity (TA), juice TSS/TA ratio, juice total sugar content, and juice reducing sugar content. Statistical analyses and graphical representations were performed using R 4.3.

## 5. Conclusions

Combining surface drip irrigation and mulching techniques into pomegranate production systems can be a valuable strategy for optimizing plant performance and maximizing crop productivity, with better performance in some vegetative and productive parameters for the less sustainable plastic mulch. The outcomes of this study have practical implications for pomegranate growers and agricultural practices. Considering the additive effects, extension specialists and farmers can explore reduced irrigation to maintain suitable plant growth and fruit yields. Additionally, the application of mulch, particularly plastic mulch, can be employed to enhance the plant performance, fruit yield, and quality. Our findings provide constructive information for developing sustainable farming practices that are able to optimize water utilization, improve fruit quality, and guarantee adequate productivity in pomegranate.

## Figures and Tables

**Table 1:** Effect of the irrigation, the mulching, and their interaction on vegetative traits (plant height, canopy circumference, and tree volume) of pomegranate plants measured at the beginning (t_0_) and the end (t_end_) of the experiment. Separately for each source of variation and within each column, means followed by a common letter are not significantly different according to the Tukey test (*p* ≤ 0.05).

Source of Variation	Plant Height (m)		Canopy Circumference (m)		Tree Volume (m^3^)	
	t_0_	t_end_	t_0_	t_end_	t_0_	t_end_
Irrigation (I)						
I100	1.72 ± 0.01 a	2.32 ± 0.02 a	4.89 ± 0.02 a	6.98 ± 0.08 a	2.18 ± 0.02 b	6.03 ± 0.18 a
I80	1.75 ± 0.02 a	2.29 ± 0.03 a	4.83 ± 0.06 a	6.85 ± 0.10 a	2.31 ± 0.03 a	5.72 ± 0.20 a
I60	1.76 ± 0.02 a	2.17 ± 0.03 b	4.97 ± 0.04 a	6.51 ± 0.09 b	2.16 ± 0.06 b	4.90 ± 0.17 b
Significance	n.s.	***	n.s.	***	*	***
Mulching (M)						
NoM	1.73 ± 0.02 a	2.20 ± 0.04 b	4.94 ± 0.05 a	6.65 ± 0.09 b	2.24 ± 0.05 a	5.18 ± 0.20 b
PM	1.74 ± 0.02 a	2.32 ± 0.03 a	4.94 ± 0.04 a	7.04 ± 0.11 a	2.25 ± 0.05 a	6.11 ± 0.24 a
OM	1.76 ± 0.01 a	2.27 ± 0.03 ab	4.81 ± 0.039 a	6.66 ± 0.08 b	2.16 ± 0.03 a	5.36 ± 0.18 b
Significance	n.s.	***	n.s.	**	n.s.	***
I × M						
Significance	n.s.	n.s.	n.s.	n.s.	n.s.	n.s.

*, **, ***, and n.s. indicate significant differences at *p* ≤ 0.05, *p* ≤ 0.01, and *p* ≤ 0.001 and not significant (*p* > 0.05) according to the two-way ANOVA, respectively.

**Table 2:** Effect of the irrigation, the mulching, and their interaction on the fruit yield, number of fruits per tree, and fruit fresh weight of pomegranate plants. Separately for each source of variation and within each column, means followed by a common letter are not significantly different according to the Tukey test (*p* ≤ 0.05).

Source of Variation	Fruit Yield (kg/Plant)	Yield Components	
		No Fruits/Plant	Fruit Fresh Weight (g/Fruit)
Irrigation (I)			
I100	13.9 ± 0.6 a	70 ± 1 a	199.2 ± 6.8 a
I80	12.3 ± 0.5 b	66 ± 2 ab	186.0 ± 4.4 b
I60	10.6 ± 0.6 c	61 ± 1 b	171.7 ± 7.6 c
Significance	***	**	***
Mulching (M)			
NoM	10.2 ± 0.5 b	63 ± 1 b	161.8 ± 5.0 b
PM	13.6 ± 0.6 a	68 ± 1 a	199.7 ± 4.9 a
OM	13.0 ± 0.5 a	66 ± 2 ab	195.5 ± 3.8 a
Significance	***	*	***
I × M			
Significance	n.s.	n.s.	***

*, **, ***, and n.s. indicate significant differences at *p* ≤ 0.05, *p* ≤ 0.01, and *p* ≤ 0.001 and not significant (*p* > 0.05) according to the two-way ANOVA, respectively.

**Table 3:** Effect of the irrigation, the mulching, and their interaction on the fruit length, diameter, volume and shape index, and rind thickness of pomegranate fruit at harvest. Separately for each source of variation and within each column, means followed by a common letter are not significantly different according to the Tukey test (*p* ≤ 0.05).

Source of Variation	Fruit Length (mm)	Fruit Width (mm)	Fruit Volume (cm^3^)	Fruit Shape Index	Rind Thickness (mm)
Irrigation (I)					
I100	81.4 ± 1.8 a	69.5 ± 0.9 a	207.2 ± 9.6 a	1.17 ± 0.01 a	4.2 ± 0.2 a
I80	76.9 ± 1.2 b	66.7 ± 1.5 ab	180.0 ± 9.5 b	1.16 ± 0.03 a	4.2 ± 0.2 a
I60	71.8 ± 0.4 c	63.9 ± 0.6 b	153.3 ± 2.8 c	1.12 ± 0.01 a	3.5 ± 0.2 b
Significance	***	**	***	n.s.	***
Mulching (M)					
NoM	73.2 ± 0.8 b	64.6 ± 0.5 a	160.1 ± 4.1 b	1.13 ± 0.01 a	3.3 ± 0.1 c
PM	79.3 ± 2.0 a	68.2 ± 1.3 a	194.8 ± 12.0 a	1.16 ± 0.01 a	4.5 ± 0.1 a
OM	77.5 ± 1.8 a	67.3 ± 1.6 a	185.6 ± 11.4 a	1.15 ± 0.03 a	4.1 ± 0.2 b
Significance	***	n.s.	**	n.s.	***
I × M					
Significance	***	n.s.	n.s.	n.s.	n.s.

**, ***, and n.s. indicate significant differences at *p* ≤ 0.01, and *p* ≤ 0.001 and not significant (*p* > 0.05) according to the two-way ANOVA, respectively.

**Table 4:** Effect of the irrigation, the mulching, and their interaction on the juice total soluble solids (TSS), pH, titratable acidity (TSS/TA), total sugars, reducing sugars, and anthocyanin content of pomegranate fruit at harvest. Separately for each source of variation and within each column, means followed by a common letter are not significantly different according to the Tukey test (*p* ≤ 0.05).

Source of Variation	TSS (°Brix)	pH	TA (%)	TSS/TA	Total Sugars (%)	Reducing Sugars (%)
Irrigation (I)						
I100	15.3 ± 0.3 a	3.24 ± 0.11 ab	0.35 ± 0.01 b	44.1 ± 2.2 b	13.71 ± 0.26 a	11.53 ± 0.15 a
I80	15.5 ± 0.2 a	3.33 ± 0.14 a	0.34 ± 0.02 c	47.0 ± 2.5 a	13.47 ± 0.36 a	11.58 ± 0.17 a
I60	14.8 ± 0.2 b	3.17 ± 0.07 b	0.37 ± 0.01 a	40.2 ± 1.9 c	13.01 ± 0.27 b	11.01 ± 0.16 b
Significance	**	*	***	***	**	***
Mulching (M)						
NoM	14.5 ± 0.2 b	2.87 ± 0.04 b	0.41 ± 0.01 a	35.3 ± 0.8 b	12.33 ± 0.16 c	10.95 ± 0.07 c
PM	15.7 ± 0.2 a	3.43 ± 0.09 a	0.32 ± 0.01 b	48.9 ± 1.4 a	14.16 ± 0.16 a	11.85 ± 0.13 a
OM	15.4 ± 0.1 a	3.44 ± 0.04 a	0.33 ± 0.01 b	47.2 ± 1.1 a	13.70 ± 0.14 b	11.32 ± 0.17 b
Significance	***	***	***	***	***	***
I × M						
Significance	n.s.	***	n.s.	n.s.	n.s.	n.s.

*, **, ***, and n.s. indicate significant differences at *p* ≤ 0.05, *p* ≤ 0.01, and *p* ≤ 0.001 and not significant (*p* > 0.05) according to the two-way ANOVA, respectively.

## Data Availability

The data supporting the findings of this study that are not already available within the article and its supplementary materials can be requested from the corresponding author (V.P.S.).

## Supplementary Materials

The following supporting information can be downloaded at: https://www.mdpi.com/article/10.3390/plants12183241/s1, Figure S1: Weekly (standard meteorological) weather data of 2019–20 at G. B. Pant University of Agriculture and Technology, Pantnagar, Uttarakhand, India. Table S1: Eigenvalue, percent of explained variance, and cumulative percent of explained variance of the eight principal components (Dim.) extracted.
