# Supplementary material for: Enhancing the Fruit Yield and Quality in Pomegranate: Insights into Drip Irrigation and Mulching Strategies
Source: Plants (Basel). 2023 Sep 12;12(18):3241. doi: 10.3390/plants12183241 (PMC10535448; doi:10.3390/plants12183241)
Supplement: Supplementary file 1 [file plants-12-03241-s001.zip › plants-2496004-supplementary.pdf]

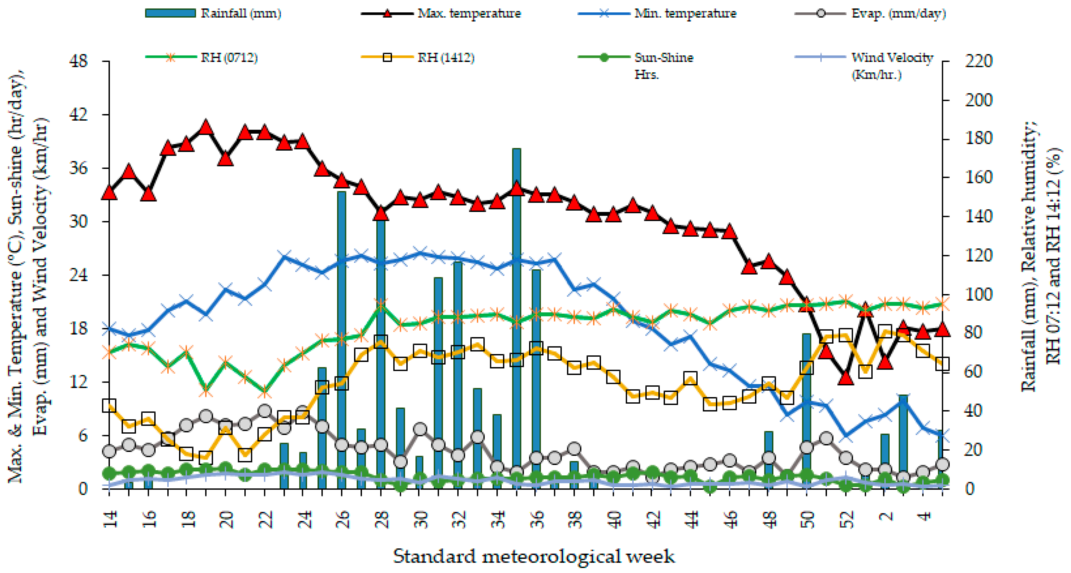

Supplementary Figure S1. Weekly (Standard meteorological) weather data of 2019-20 at G. B. Pant University of Agriculture and Technology, Pantnagar, Uttarakhand, India.

Supplementary Table S1. Eigenvalue, percent of explained variance, and cumulative percent of explained variance of the eight principal components (Dim.) extracted

Principal component	Eigenvalue	Variance explained (%)	Cumulative variance explained (%)
Dim. 1	13.56	67.80	67.80
Dim. 2	2.63	13.15	80.95
Dim. 3	2.41	12.05	93.00
Dim. 4	0.69	3.45	96.45
Dim. 5	0.33	1.65	98.10
Dim. 6	0.2	1.00	99.10
Dim. 7	0.15	0.75	99.85
Dim. 8	0.03	0.15	100.00
